# One year results of a pilot study on stepwise defocus incorporated multiple segment spectacle wear for myopia prevention in preschool children with premyopia

**DOI:** 10.1038/s41598-026-52489-5

**Published:** 2026-05-13

**Authors:** Hsin-Yu Yang, Der-Chong Tsai, Chiao-Yu Wang, Yu-Chieh Yang, Chia-Wei Lee, Pei-Wei Huang, Mong-Ping Shyong, Shih-Hwa Chiou, Tzu-Tang Lin, Yen-Lin Chen, Nai-Wei Hsu

**Affiliations:** 1 Department of Ophthalmology, Yuanshan and Suao Branch, Taipei Veterans General Hospital, Yilan, Taiwan; 2 Myopia Prevention and Care Center, Department of Ophthalmology, National Yang Ming Chiao Tung University Hospital, Yilan, Taiwan; 3 Institute of Clinical Medicine, National Yang Ming Chiao Tung University, Taipei, Taiwan; 4 Faculty of Medicine, National Yang Ming Chiao Tung University School of Medicine, Taipei, Taiwan; 5 Community Medicine Research Centre and Institute of Public Health, National Yang Ming Chiao Tung University, Taipei, Taiwan; 6 Department of Ophthalmology, Taipei Veterans General Hospital, Taipei, Taiwan; 7 Department of Ophthalmology, Fu Jen Catholic University Hospital, Fu Jen Catholic University, New Taipei City, Taiwan; 8 School of Medicine, College of Medicine, Fu Jen Catholic University, New Taipei City, Taiwan; 9 Department of Ophthalmology, Lotung Poh-Ai Hospital, Lo-Hsu Medical Foundation, Yilan, Taiwan; 10 Institute of Pharmacology, National Yang Ming Chia Tung University, Taipei, Taiwan; 11 Public Health Bureau, Yilan County, Taiwan; 12 Division of Cardiology, Department of Internal Medicine, National Yang Ming Chiao Tung University Hospital, Yilan, Taiwan

**Keywords:** Myopia, Myopic-defocus spectacles, Premyopia, Preschoolers, Spherical equivalent, Diseases, Health care, Medical research

## Abstract

This study assessed the feasibility, acceptability, and preliminary efficacy of stepwise defocus incorporated multiple segment (DIMS) plano spectacle wear by preschoolers with premyopia and normal uncorrected visual acuity (UCVA). This single-arm, prospective pilot study included 23 children with premyopia (mean age 5.4 years) who were identified from a community screening program in Taiwan. A stepwise DIMS spectacle regimen was implemented: part-time wear during near work before school entry followed by full-time wear after starting school. The primary outcome was the change in the spherical equivalent (SE) and the secondary outcome was axial length (AXL) elongation after 1 year. The mean SE change after 1 year was + 0.08 dioptres (0.35), whereas the mean AXL elongation was + 0.22 mm (0.12). None of the participants developed myopia. During the preschool stage, 91.3% had good adherence to part-time wear. However, adherence dropped to 52.2% during full-time wear after school entry. The majority of the participants (73.9%) had physiological AXL growth. Visual symptoms were minimal and the caregivers reported high acceptance. Stepwise use of the DIMS plano spectacles was well-tolerated and associated with stable refractive outcomes and physiological AXL elongation among preschoolers with premyopia. Larger, controlled studies in future are warranted to validate these findings and optimise intervention strategies.

**Supplementary Information:**

The online version contains supplementary material available at 10.1038/s41598-026-52489-5.

## Introduction

Myopia prevalence has increased in East Asia, where high myopia poses serious ocular risks and healthcare burdens. Early-onset myopia in childhood strongly predicts high myopia in adulthood, making its prevention a priority^1^.

In 2019, the International Myopia Institute introduced the term ‘premyopia’ to describe a refractive state between > − 0.50 dioptres (D) and ≤ 0.75 D in children with other risk factors for myopia^2,3^. Longitudinal research has consistently identified baseline refractive errors as the strongest predictor of myopia onset in children without myopia^4–6^. Hence, young children with premyopia should be the primary focus of early myopia prevention strategies. However, apart from behavioural strategies promoting outdoor time, currently, there is no consensus regarding preventing the progression from premyopia to myopia using pharmacological (low-dose atropine) or optical (peripheral defocus spectacles) interventions.

Low-dose atropine has shown efficacy in East Asian premyopia trials, with LAMP2 identifying 0.05% as the most effective concentration for preventing myopia onset^7–10^. Intermediate doses (e.g., 0.02%) may offer a better balance between efficacy and tolerability^11^. Evidence in Western premyopic populations is lacking; however, studies in Western children with established myopia suggest that 0.01% atropine provides only modest benefit^12–14^. Overall, optimal dosing appears context-dependent.

Peripheral defocus spectacles offer a non-pharmacological alternative without atropine’s side effects. For Chinese children, highly aspherical lenslets (HAL) worn > 30 h/week reduced axial elongation in a dose-dependent manner^15^. However, maintaining compliance among preschoolers with premyopia and good uncorrected visual acuity (UCVA) is challenging due to high outdoor activity and low near-work demands.

To address this issue, we implemented a stepwise defocus incorporated multiple segment (DIMS) spectacle regimen for preschoolers with premyopia, which employs part-time and full-time wear before and after school entry, respectively. DIMS lenses, with + 3.50 D defocus segments around a clear central zone, slow myopia progression; however remain understudied for prevention^16,17^.

This pilot study evaluated the acceptability of DIMS plano spectacles by preschoolers with normal UCVA and their 1-year effects on axial length (AXL) and spherical equivalent (SE) under this regimen.

## Methods

### Study design

This pilot study was approved by the Institutional Review Board of National Yang Ming Chiao Tung University Hospital (NYCUHIRB No. 2023A015). Written informed consent was obtained from the participating children and their parents or guardians. This study adhered to the tenets of the Declaration of Helsinki.

To determine the feasibility and efficacy of slowing the SE myopia shift in children with premyopia around the school entrance age, we conducted this pilot study at the National Yang Ming Chiao Tung University Hospital.

### Participant recruitment and eligibility

Participants were recruited from a myopia prevention and improvement program^18^. Preschoolers with premyopia identified via cycloplegic refraction were invited to join with parental consent.

Inclusion criteria were: age 5–6 years (senior kindergarten), cycloplegic SE of the less hyperopic eye between <  + 1.00 D and > − 0.50 D, astigmatism ≤ 1.5 D in both eyes, anisometropia ≤ 1.50 D, monocular UCVA of 6/7.5 or better in both eyes, and signed informed consent.

Exclusion criteria comprised: strabismus or ocular motility disorders, any ophthalmic or systemic condition affecting visual function or refractive development, and prior use of atropine or other myopia control treatments.

### Intervention

In this study, the central optical zone of the DIMS lenses was non-refractive because all the participants had normal UCVA. Owing to lower academic and environmental stress in preschool-aged children, the participants with premyopia initially wore lenses part-time, mainly during near-work at home and on weekends or holidays. Full-time wear was required upon either elementary school entry or a myopic SE shift of ≥ 0.50 D, whichever came first. Unlike children with myopia who are prescribed to full-time wear, these participants with premyopia were not required to wear DIMS spectacles during daytime hours in kindergarten. After transitioning to full-time use, lenses were worn continuously, except during sleep and bathing, until study completion. This stepwise, part-time wearing strategy was guided by several considerations. Outdoor activity is encouraged and academic demands are relatively low in kindergarten, whereas near work and screen use predominantly occur at home. Part-time wear may facilitate adaptation to DIMS spectacles in premyopic preschoolers before transitioning to full-time wear in elementary school, while also allowing parents to monitor early adaptation.

At dispensing and each follow-up, study staff provided face-to-face instructions to participants and caregivers regarding spectacle care and adherence. If cycloplegic SE reached ≤ − 1.00 D in either eye, DIMS lenses were updated to maintain a corrected visual acuity of 6/7.5 or better.

### Follow-up schedule

In addition to the baseline visit, all participants underwent follow-up visits at 3, 6, 9, and 12 months. Most participants entered elementary school after the first 9 months of follow-up. The differences in the variables of interest between baseline and each follow-up visit were analysed.

### Eye examinations

The participants underwent eye exams at baseline and every 3 months, including UCVA, refractive error (pre-/post-cycloplegia), AXL, external and fundus exams, ocular alignment, motility, and sub foveal choroidal thickness (SFCT).

### Visual acuity and autorefraction

Refractive error was measured with an autorefractor (Topcon KR-1, Tokyo, Japan), averaging 3 readings. UCVA was tested monocularly at 6 m. Distance and near visual acuity (NVA, at 33 cm, 20° below horizontal) were assessed with DIMS spectacles at each follow-up.

### Cycloplegia and cycloplegic autorefraction

Cycloplegia was induced using 1% tropicamide instilled every 10 min for three doses, with a fourth dose administered if the pupillary light reflex persisted. Cycloplegic autorefraction was performed only after confirming complete cycloplegia, and the final value was obtained by averaging three consistent readings per eye using the Topcon KR-1 autorefractor.

### Ocular biometry and choroidal thickness

AXL, lens thickness, keratometry, and anterior chamber depth were measured with Lenstar LS 900. SFCT was assessed by optical coherence tomography (Optovue), averaging vertical and horizontal foveal scans. SFCT was assessed using optical coherence tomography (OCT; Optovue), with measurements obtained from both vertical and horizontal foveal scans and averaged for analysis. All examinations were conducted during daytime hours to minimize the effects of diurnal variation. Measurements were independently performed by three experienced observers who were masked to participant identity, clinical data, and visit sequence. If the inter-observer difference was < 25 μm, the mean value was used; otherwise, a consensus measurement was obtained.

### Questionnaire survey and adherence assessment

At the 1-week visit, the participants completed visual acuity testing and reported visual symptoms and lens acceptability. A standardised questionnaire rated 14 symptoms (0–4 scale from never, rarely, sometimes, usually, and always according to the frequency), administered orally with caregiver assistance. The caregivers answered 2 acceptability questions and kept diaries of daily activities and spectacle use.

Adherence was assessed using a predefined time-block system to standardize caregiver reporting. Each day was divided into three blocks (morning, afternoon, and after-school/evening), totalling 21 blocks per week. During the preschool period, spectacles were prescribed for 11 blocks/week (weekday after-school and all weekend blocks), whereas after school entry, participants were instructed to wear spectacles during all 21 blocks/week. Good adherence was defined as ≥ 75% completion of prescribed blocks.

### Outcome analysis

The primary outcome was the 1-year myopic shift in SE refraction, calculated as the difference between SE values at baseline compared with those at 12 months afterwards. SE was selected as the primary outcome due to its direct clinical relevance in premyopia, where the key objective is to prevent or delay the onset of myopia (typically defined as SE ≤  − 0.50 D). The secondary outcome was the change in mean AXL over the same period. AXL data were evaluated using the age-matched myopia control (AMMC) framework (Haag-Streit, Switzerland) to classify growth rates as slow/physiological, moderate, or fast^17^. AMMC is a simplified nomogram plotting AXL growth rate against age and categorized growth into three color-coded zones. The green zone represents physiological AXL growth, with an upper boundary extended by + 25% to account for measurement variability. The yellow zone indicates moderately excessive growth, defined as + 25% to + 50% above the age-specific physiological AXL growth rate. The red zone denotes highly excessive growth, exceeding + 50% above the age-matched physiological rate. Sex-specific differences in AXL growth patterns were accounted for by analyzing males and females separately^17^. The nomogram is based on longitudinal axial length growth data from emmetropic children derived primarily from large-scale European cohorts^19,20^.

Exploratory outcomes included changes in mean SFCT, mean lens thickness, mean corneal curvature, and near-work and outdoor activity levels during the study.

### Statistical analyses

Only the eye with lower SE was analysed for each participant. Continuous variables were summarised as mean (SD), and categorical as counts (%). Group differences used Wilcoxon rank sum or Chi-square tests, and paired data used Wilcoxon signed-rank or McNemar’s test. Analyses were performed using SPSS 26 (IBM Corp., Armonk, NY, USA), with significance set at *P* < 0.05.

## Results

Of the 24 eligible participants recruited in this pilot study, 1 withdrew due to inability to adhere to the follow-up schedule, resulting in a final cohort of 23 participants (11 boys [47.8%]; mean age, 5.4 years [0.49]) being included in the analyses. Among them, both parents had myopia for 15, 7 had a single parent with myopia, and 1 did not have parents with myopia. At baseline, the mean SE refraction was + 0.39 D (0.32), mean AXL was 22.49 mm (0.63), and mean SFCT was 353.0 μm (56.5). All the participants had UCVA of 1.0 in both eyes, and the mean NVA while wearing DIMS lenses was 0.995 (0.02).

### Adaptation and acceptance of DIMS plano spectacles

**Fig. 1:**
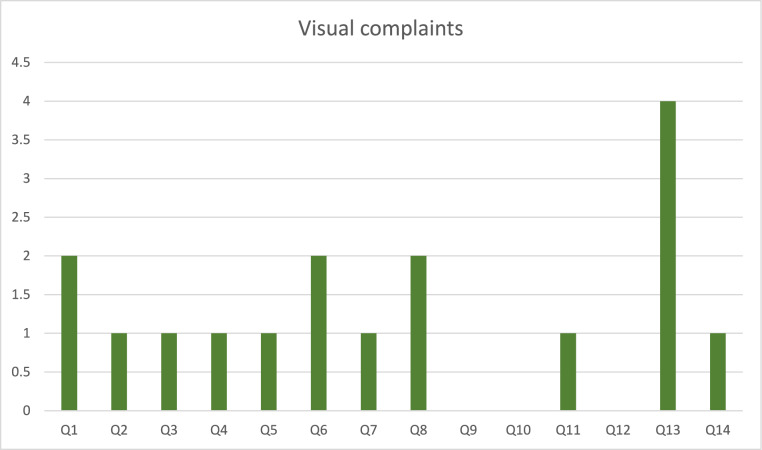
Visual complaints after 1 week of wearing defocus incorporated multiple segment spectacles. Q1: visual strain; Q2: headache; Q3: dizziness; Q4: diplopia; Q5: nausea; Q6: photophobia; Q7: blurry vision; Q8: lens position adjustment needed; Q9: darkness; Q10: colour change; Q11: visual shadow; Q12: size change in vision; Q13: inconvenience in daily activities at home; and Q14: inconvenience in daily activities at school. Y axis: number of cases reported.

After 1 week of wearing DIMS plano spectacles, the most commonly reported visual complaint was inconvenience during daily activities at home, as conveyed by 17.4% of the participants. Other reported complaints included visual strain (8.7%), photophobia (8.7%), and the need to adjust lens position (8.7%). The overall frequency and severity of symptoms were graded as “rarely” for all the symptoms. (Fig. 1). All caregivers expressed willingness for their children to continue wearing DIMS plano spectacles in response to both acceptability questions.

### Changes in SE refraction, AXL, and SFCT during the preschool stage (from baseline to month 9)

All 23 preschool participants entered elementary school after the first 9 months of the follow-up period and remained without myopia during the preschool stage; a part-time wearing approach was adopted. Between baseline and month 9, the mean SE refraction remained stable, shifting slightly from 0.386 D (0.32) to 0.473 D (0.27) (*P* = 0.265). During the preschool follow-up period, a mean AXL elongation of 0.15 mm was observed (from 22.49 mm to 22.64 mm, p < 0.001). The mean SFCT remained stable, changing from 353 μm (56.5) to 349.8 μm (51.2) (*P* = 0.754). None of the participants with premyopia exhibited a fast myopic SE shift during the premyopic stage.

### One-year changes in SE refraction, AXL, lens thickness, corneal curvature and SFCT

**Fig. 2:**
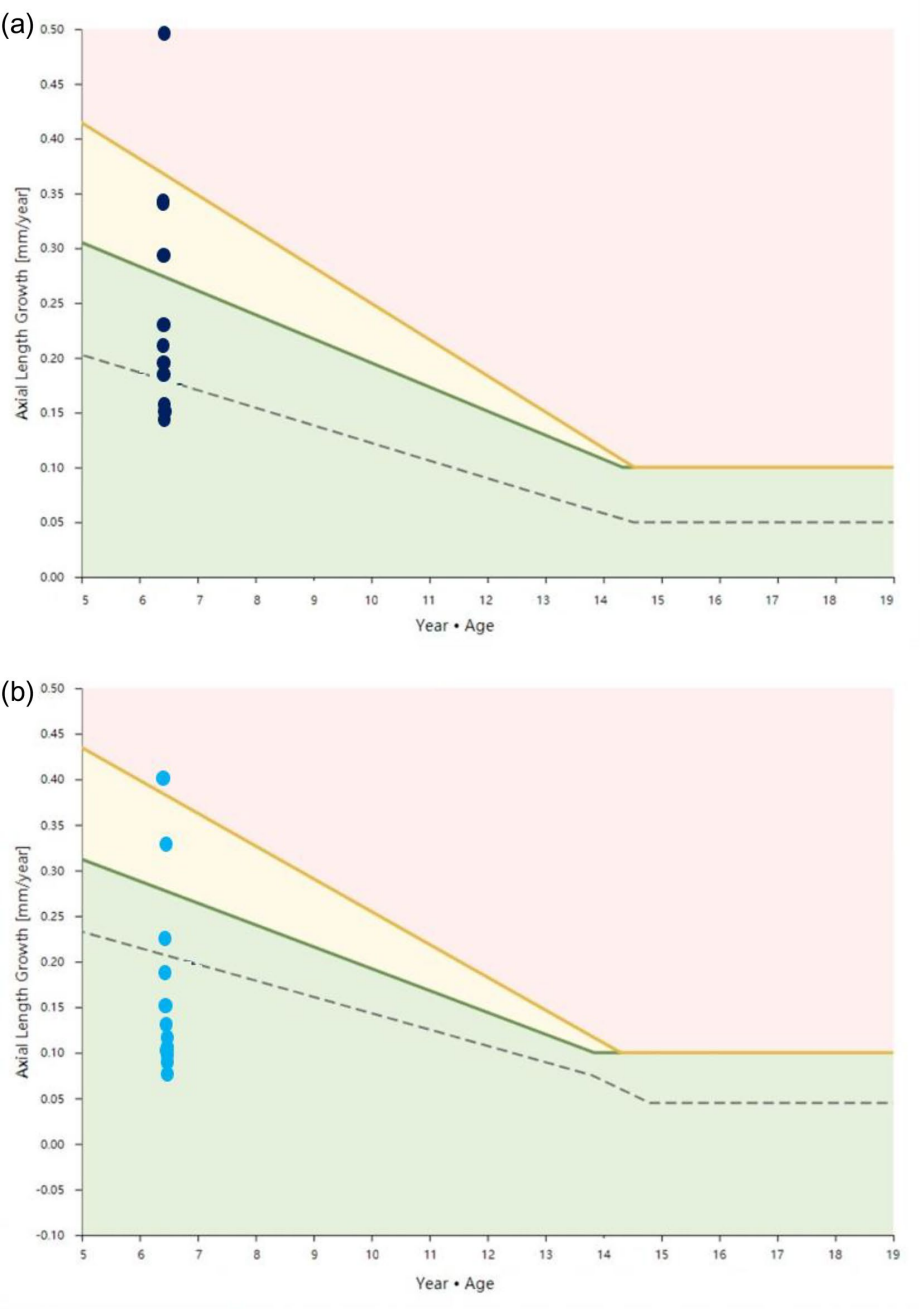
Annual axial length growth rate at 1-year follow-up, evaluated using an age-matched myopia control (AMMC) system, for (**a**) male and (**b**) female participants. The green zone represents physiological growth (with the upper limit extended by + 25% to account for measurement variability), the yellow zone indicates moderately excessive growth (+ 25% to + 50% above physiological), and the red zone denotes highly excessive growth (> + 50% above physiological). Males and females were analyzed separately to account for sex-specific differences in growth patterns.

Table 1 presents the distributions of SE refraction, AXL, lens thickness, corneal curvature and SFCT of each follow-up visit over 1 year. Among all 23 participants, the mean AXL showed elongation, increasing from 22.49 mm at baseline to 22.71 mm at one year (mean change: + 0.224 mm; p < 0.0001). Despite this axial growth, the SE refraction remained stable, shifting from + 0.39 D to + 0.47 D (mean change: + 0.082 D; median: 0 D; p = 0.274). This stability in refractive error, despite axial elongation, may be attributed to compensatory changes in internal ocular structures. Specifically, lens thickness decreased significantly from 3.61 mm to 3.57 mm (mean change: − 0.042 mm; p = 0.045). In contrast, corneal curvature remained relatively stable, with a non-significant change from 43.52 D to 43.71 D (mean change: + 0.193 D; p = 0.297). Additionally, mean SFCT showed a negligible decrease of − 2.5 μm (from 353.0 μm to 350.5 μm; median change: + 1.0 μm; p = 0.631). Myopia was not observed in any of the participants during the 1-year follow-up. The rate of SE change shifted from + 0.0097 D/month during the first 9 months prior to elementary school entry to − 0.002 D/month in the final 3 months following school entry. Although the change did not reach significance (*P* = 0.870), it indicates a stable trend of SE. Similarly, AXL elongation varied across these intervals, increasing by 0.017 mm/month initially and accelerating to 0.024 mm/month thereafter (*P* = 0.170). Using the AMMC framework for AXL elongation analysis, there were 2 (8.7%) participants (1 girl and 1 boy) categorised as having high AXL growth, 4 (17.4%, 1 girl and 3 boys) as having moderate AXL growth, and 17 (73.9%) as having low/tolerable AXL growth (Fig. 2).

**Table 1:** One year change of ocular parameters after DIMS wearing.

Parameter	Baseline (Mean ± SD)	1 Year (Mean ± SD)	Mean Change	P-value*
Axial length (mm)	22.489 ± 0.626	22.713 ± 0.669	+ 0.224	< 0.0001
Corneal curvature (D)	43.520 ± 1.507	43.713 ± 1.424	+ 0.193	0.2973
Lens thickness (mm)	3.613 ± 0.178	3.571 ± 0.179	− 0.042	0.0447
Spherical equivalent (D)	0.386 ± 0.322	0.467 ± 0.350	+ 0.082	0.2743
SFCT (µm)	352.978 ± 56.503	350.500 ± 53.313	− 2.478	0.6309

### Adherence to the DIMS plano spectacle wearing

During the part-time wearing phase before school entry, 91.3% of the 23 participants demonstrated adherence rates exceeding 75%. Conversely, only 52.2% maintained adherence rates above 75% during the full-time wearing phase after school entry. The proportion of participants with good adherence was substantially higher in the part-time phase compared with the full-time phase (*P* < 0.001).

### Myopia-related lifestyle/behaviours outside of school

Based on parent-recorded diaries during the study period, the mean duration of outdoor activity was 32.2 min/day on weekdays and 162.4 min/day on weekends. On weekdays, children spent an average of 36.3 min/day on reading and writing homework, and 60.8 min on screen-based devices. On weekends, the corresponding averages were 33.7 and 113.3 min for homework and screen-based devices, respectively. Significant correlations were not found between 1-year changes in SE refraction or AXL and at-home or weekend lifestyle/behavioural factors. (supplementary table 1).

## Discussion

In this one-year pilot study, we demonstrated that children with premyopia with normal UCVA can generally tolerate wearing DIMS plano spectacles and may potentially benefit from their use in slowing the myopic SE shift, even when worn part-time for near-work before entering elementary school. Most of the preschool participants (91.3%) had good adherence to the part-time wearing regimen. During the preschool follow-up period, a mean AXL elongation of 0.15 mm was observed. However, mean SE remained relatively stable (+ 0.087 D), and no incident myopia cases were detected before school entry. These preliminary findings suggest that part-time use of DIMS plano spectacles is a feasible approach and may be associated with relative refractive stability in preschool children with premyopia. However, given the pilot design and absence of a control group, these observations should be interpreted with caution and require confirmation in future randomized controlled studies.

The rationale for the stepwise, part-time wearing strategy in our pilot study is based on several considerations. First, outdoor activities are actively promoted in kindergartens, and the educational burden is generally lower than in elementary school. Second, preschool children are more likely to engage in near work and use screen-based electronic devices at home rather than in kindergarten settings. Third, prior to transitioning to full-time wear after entering elementary school, a part-time wearing approach may facilitate adaptation to DIMS spectacles in premyopic preschoolers with normal vision. Taken together, this strategy may provide targeted intervention during higher-risk periods while maintaining feasibility in real-world settings.

Although visual complaints were minimal, full-time wear of plano DIMS spectacles was difficult to implement in premyopic preschoolers with normal UCVA, particularly during physical activities. In contrast, part-time wear during near work at home was well accepted, despite minor initial inconveniences. Adherence was higher during the preschool part-time phase than after transition to full-time wear in elementary school.

Although the part-time regimen (11 blocks/week) is more tolerable for preschoolers, its efficacy remains a key concern. Notably, participants with great adherence could achieve an estimated wear time of approximately 44 h/week (4 h × 5 weekdays and 12 h × 2 weekend days), exceeding the > 30 h/week threshold reported for HAL spectacles to reduce axial elongation^15^. No incident myopia or rapid SE progression was observed, and most participants (73.9%) demonstrated physiological AXL growth over 1 year. These findings suggest that part-time wear of plano DIMS spectacles appears safe before school entry and may confer a potential preventive effect against myopia in premyopic preschoolers.

This favourable outcome of part-time DIMS wear before elementary school entry may be partially attributed to the Yilan myopia prevention and vision improvement program (YMVIP)—a health promotion initiative in Yilan County, Taiwan—that encourages outdoor activities among kindergarten children. Increased time spent outdoors is a well-established protective factor against the development of childhood myopia, particularly in younger children^6^. Further studies are warranted to investigate the effect of daytime outdoor activity on DIMS plano spectacle wearing time across different age groups.

This pilot study required all the participants to shift from part-time to full-time wearing of DIMS plano spectacles after entering elementary school, regardless of their prior SE changes. Although outdoor activity is promoted in Taiwan’s elementary schools through the public health initiative Tien-Tien Outdoor 120 program^6^, the learning environment and academic pressure differ substantially between elementary schools and kindergartens. A nationwide survey in Taiwan conducted in 2017 revealed that the prevalence of myopia in first-grade schoolchildren (19.8%) was more than twice that of kindergarteners (9.0%)^21^. In the present study, the mean AXL growth rate increased after school entry compared to before, and the mean SE change also shifted in a myopic direction.

Despite the requirement for full-time wear, adherence during the final 3 months was much lower than that during the initial 9-month follow-up period, which may partially explain the diminished treatment effect observed after school entry. In a clinical trial investigating the effects of HAL spectacles on changes in SE and AXL in children with low hyperopia aged 6–9.9 years, the participants were instructed to wear the spectacles for at least 4 h/day. Notably, only those with HAL spectacle wear exceeding 30 h/week showed significantly slower AXL elongation^15^. In another study that used optical technical of guiding emmetropization (OTOGE) lenses, a type of peripheral defocus spectacle lens, for myopia prevention in children without myopia aged 5–8 years, a minimum wear time of 10 h/day was mandated; after 1 year, both SE myopic shift and AXL elongation were significantly lower in the OTOGE group compared with the control group^22^. These findings underscore the importance of extended wear time for peripheral defocus spectacle lenses among school-aged children, who are likely to engage in more near-work during elementary school than that in kindergarten.

Although the myopia control effect of DIMS spectacle lenses has been well-documented among children with myopia^16,17^, their role in myopia prevention remains unclear. In a pivotal clinical trial involving 183 Chinese children aged 8–13 years with myopia ranging from − 1.0 D to − 5.0 D, the 1-year mean SE change was − 0.17 D (0.05) in the DIMS spectacle group compared with − 0.55 D (0.04) in the single vision spectacle group. The corresponding mean AXL change was + 0.11 D (0.02) and + 0.32 D (0.02), respectively^17^. When these 1-year AXL data were evaluated using the AMMC framework, 65% of eyes in the DIMS group exhibited physiological levels of AXL growth, whereas 28% showed highly excessive elongation^17^. Conversely, the present study found that the 1-year mean AXL change in 23 preschoolers with premyopia aged 5–6 years was + 0.22 D (0.12), approximately twice the rate observed in the schoolchildren with myopia of the aforementioned DIMS group. This difference may be attributed to the age gap between participants in the 2 studies; the mean age at enrolment was 10.19 years in the DIMS group compared with 5.4 years in the present study. AXL growth rate is age-dependent and generally declines with increasing age^17^. Nevertheless, participants in this pilot study were more likely to exhibit physiological AXL elongation than schoolchildren with myopia (73.9% vs. 65%) and were less likely to demonstrate highly excessive AXL growth (8.7% vs. 28%). This may help explain the refractive stability observed in this cohort with premyopia because the mean SE did not reveal a myopic shift over the 1 year. These findings suggest that early intervention with DIMS spectacle lenses may help maintain AXL growth within the physiological range in young children at risk of developing myopia.

To further investigate this apparent dissociation between AXL and SE change, we analyzed changes in lens thickness and corneal curvature over one year. Lens thickness decreased significantly while corneal curvature showed no apparent change. This finding supports the presence of compensatory lens thinning, whereby reduction in crystalline lens thickness may offset axial elongation and contribute to the maintenance of refractive stability in premyopic children.

This single-arm pilot study has a few limitations. First, the small sample size and absence of a control group make it difficult to directly evaluate the myopia prevention effects of DIMS spectacle lenses in preschoolers with premyopia. However, the physiological AXL growth range provided by the AMMC framework can serve as a reference. Additionally, refractive changes observed in our previous longitudinal study provide a useful comparative baseline. This study, which included 742 primary-grade schoolchildren in Yilan identified as having premyopia during preschool YMVIP screenings, reported an SE progression rate of − 0.19 D/year (0.39) and a myopia incidence density of 14.8% per person-year^18^. Notably, these references do not constitute a true control group, and such comparisons should therefore be interpreted with caution. Second, adherence to the spectacle-wearing regimen was assessed using caregiver-administered diary logs and was defined based on the proportion of predefined time blocks rather than actual wearing time. Although practical, this subjective method raises concerns about the possibility of reporting bias, including social desirability and recall bias, and accuracy of tracking actual wearing time. Last, accommodation and convergence were not assessed. Although residual accommodation may not have been fully eliminated, cycloplegic refraction using 1% tropicamide was consistently applied at baseline and follow-ups to reduce its influence on SE measurements.

This single-arm pilot study demonstrated that most preschoolers with premyopia who followed a stepwise DIMS plano spectacle regimen exhibited minimal mean SE changes and AXL elongation within physiological limits over a period of 1 year. During the preschool stage, part-time wear during near work was well-tolerated, and favourable refractive outcomes were achieved without requiring prolonged daily use. These findings support the potential of early DIMS spectacle intervention in delaying myopia onset. However, given the pilot design and lack of a control group, these findings should be interpreted with caution. Randomized controlled trials are needed to validate the results and refine integrated strategies for myopia prevention.

## Supplementary Information

Supplementary Information.

## Data Availability

The datasets used and/or analysed during the current study available from the corresponding author on reasonable request.
