# Supplementary material for: One year results of a pilot study on stepwise defocus incorporated multiple segment spectacle wear for myopia prevention in preschool children with premyopia
Source: Sci Rep. 2026 May 13;16:21895. doi: 10.1038/s41598-026-52489-5 (PMC13365823; doi:10.1038/s41598-026-52489-5)
Supplement: Supplementary file 1 — Supplementary Information. [file 41598_2026_52489_MOESM1_ESM.docx]

**Supplementary table 1:** Correlation between lifestyle factors and 1-year changes in SE and axial length

| **Variable** | **SE change (r)** | **p-value** | **AXL change (r)** | **p-value** |
| --- | --- | --- | --- | --- |
| Q1 (weekday outdoor) | -0.01 | 0.98 | -0.24 | 0.27 |
| Q2 (weekday homework) | 0.27 | 0.20 | 0.00 | 1.00 |
| Q3 (weekday mobile device) | -0.10 | 0.64 | -0.18 | 0.42 |
| Q4 (weekday computer/video games) | 0.02 | 0.95 | -0.25 | 0.25 |
| Q5 (weekday TV) | 0.11 | 0.62 | -0.23 | 0.29 |
| Q6 (weekend outdoor) | -0.15 | 0.49 | -0.28 | 0.19 |
| Q7 (weekend homework) | 0.28 | 0.20 | -0.04 | 0.85 |
| Q8 (weekend mobile device) | 0.07 | 0.76 | -0.20 | 0.35 |
| Q9 (weekend computer/video games) | -0.02 | 0.93 | 0.02 | 0.93 |
| Q10 (weekend TV) | 0.11 | 0.61 | -0.18 | 0.40 |

**Pearson correlation analyses** were conducted, and the corresponding **correlation coefficients (r) and p-values** have been calculated for all variables
